# Kothon: A large-scale dataset for machine translation of the Chittagonian and Sylheti dialects into standard Bangla

**DOI:** 10.1016/j.dib.2026.112789

**Published:** 2026-04-17

**Authors:** Md. Atique Faisal, Farhan Sadaf, Dipta Chowdhury, H.M. Azrof, Monojit Paul Tanmay

**Affiliations:** Department of Computer Science and Engineering, Khulna University of Engineering & Technology, Khulna 9203, Bangladesh

**Keywords:** Natural language processing, Machine translation, Dialect, Chittagonian, Sylheti, Standard Bangla language

## Abstract

Chittagonian and Sylheti are two major and complex Bengali dialects spoken by over 24 million Bengali speakers. However, their well-written forms are becoming increasingly rare, putting them at risk of extinction. As these dialects differ significantly from Standard Bangla, they often create communication barriers for non-dialectal speakers. Despite this, very few research efforts have been made to address the issue. Existing resources are limited to small datasets, which are insufficient for effective preservation of dialects. To bridge this gap, this study focuses on the creation and evaluation of large-scale parallel corpora for the Chittagonian-Bangla and Sylheti-Bangla translation. A total of 8000 Chittagonian and 9300 Sylheti sentences were collected and annotated by five native dialect-speaking annotators. Standard Bangla sentences were gathered from open-source resources, novels, and existing datasets, complemented with text scanning of printed books. A custom web-based annotation tool was developed to aid the annotation process. The quality and reliability of the datasets were also ensured through a rigorous validation process involving independent native speakers, who reviewed translations. This dataset serves as a valuable resource for advancing research in Bengali language processing and supporting the creation of intelligent systems that help preserve dialects and promote digital communication.

Specifications Table

**Table:** 

Subject	Computer Sciences
Specific subject area	Translation dataset on Chittagonian and Sylheti dialects into Standard Bangla
Type of data	Table (Text/String).
Data collection	The dataset was built using open-source materials collected from Bangla novels, online resources, and manual translations conducted with the help of native Chittagonian and Sylheti speakers. Printed texts were scanned using Google Lens, and data were cleaned with regular expressions. A Gradio-based web interface on Hugging Face was used for manual annotation. Fuzzy clustering was applied to detect spelling errors and normalize similar words. After a rigorous validation process, the final validated data were stored in XLSX format.
Data source location	Khulna University of Engineering & Technology, Khulna-9203, Bangladesh
Data accessibility	Repository name: Mendeley Data Data identification number: 10.17632/2fv6vf9v2z.3 Direct URL to data: https://data.mendeley.com/datasets/2fv6vf9v2z
Related research article	None

## 1 Value of the Data

• This dataset offers an important large-scale resource for two major low-resource Bengali dialects, Chittagonian and Sylheti. These dialects are quite different from Standard Bangla. Although spoken by about 8.4% of Bengali people, these dialects are currently underrepresented on digital platforms, with the risk of gradual shifting. By providing a parallel corpus of 8000 Chittagonian and 9300 Sylheti sentences aligned with Standard Bangla, the dataset fills a significant gap in available linguistic resources.
• The dataset enables a wide range of NLP research and practical applications. For example, researchers can use it to build and evaluate baseline Neural Machine Translation (NMT) models for Chittagonian↔Bangla and Sylheti↔Bangla, or to compare different architectures such as RNNs, Transformers, and pretrained models. It can also support dialect adaptation tasks for large language models (LLMs), where LLMs are fine-tuned or prompted to understand dialect-specific structures. Additional use cases include dialect identification, automatic speech recognition (ASR) support, text-to-speech, and linguistic analysis of dialectal features.
• Beyond technology, the dataset plays a vital role in preserving the cultural and linguistic identity of Chittagonian and Sylheti speakers. It supports the development of inclusive digital tools that represent these communities and creates a base for future work in dialectal Bangla NLP, ensuring these languages are not lost to modernization.
• The dataset is carefully built through a rigorous process of data collection, preprocessing, and multi-stage human validation. With native speakers directly involved in annotation and error correction, the quality and reliability of the resource are ensured. This approach can also serve as a practical model for building datasets in other low-resource or dialectal languages.
• Finally, by making the dataset freely accessible, it encourages long-term collaboration, research, and innovation in language technology for under-resourced languages. This openness ensures that the resource can be updated, expanded, and applied in future studies.

## 2 Background

Bengali is spoken by about 285 million people around the world, making it one of the most widely used languages [1]. The Bengali dialect is classified into five groups with more than twenty regional variations [2]. Among them, Chittagonian and Sylheti stand out, with around 13 million and 11 million speakers respectively [3]. These dialects are unique in their own ways.

Chittagonian belongs to the Bengali-Assamese branch of the Indo-Aryan languages and is notable for its distinct phonetic and morphological features [4]. Chittagonian is highly distinguishable from Standard Bengali and other dialects through its phonetic and morphological features [4]. While the Chittagonian dialect shares the basic vowels and consonants of Standard Bangla, it features distinctions in its sound system and displays considerable variance in morphemes [4]. Unlike Standard Bangla, Chittagonian has no written form, and its differences make it difficult for non-native dialect speakers to understand.

Sylheti dialect is an Eastern Indo-Aryan language, part of the Bengali-Assamese continuum [5]. Standard Bangla and Sylheti are closely related as they are both to Assamese. People of Sylhet, Moulvibazar, Sunamgonj, and Hobigonj in Bangladesh and Cachar, Hailakandi, and Karimganj in the state of Assam speak in the Sylheti dialect. Around 95% of the UK's Bangladeshi-origin community traces its roots to the Sylhet region [5]. Its own unique historical script, Sylheti Nagri, has a distinct literary tradition [5]. However, the young generation is not quite familiar with this script, and in different regions people speak in different tones. Modern Sylheti dialect shares Bangla alphabet and is very close to standard Bangla, but there is distinguishable variance in phonetic and morphological features [5].

Today, these dialects face a decline as many people, especially younger generations, prefer Standard Bangla for education, media, and communication. This ongoing shift poses a risk to the preservation of them, despite their rich cultural and historical significance. Very few works have been done on these two prominent dialects. Although datasets such as ChatgayyaAlap [6], Vashantor [7] and ONUBAD [8] have gained popularity, their scale remains insufficient to meet the requirements of modern NLP advancements (Table 3). In different region, people speak in different tones for the same dialect. As their well-written form is rare, it poses additional difficulty in dialectal standardization. Driven by this urgent need to protect linguistic diversity and cultural identity, this study focuses on developing large-scale parallel corpora for Chittagonian-Bangla and Sylheti-Bangla translations. The aim is to create a strong foundation that can support future research and technological progress in these less-represented language varieties.

## 3 Data Description

**Fig. 1:**
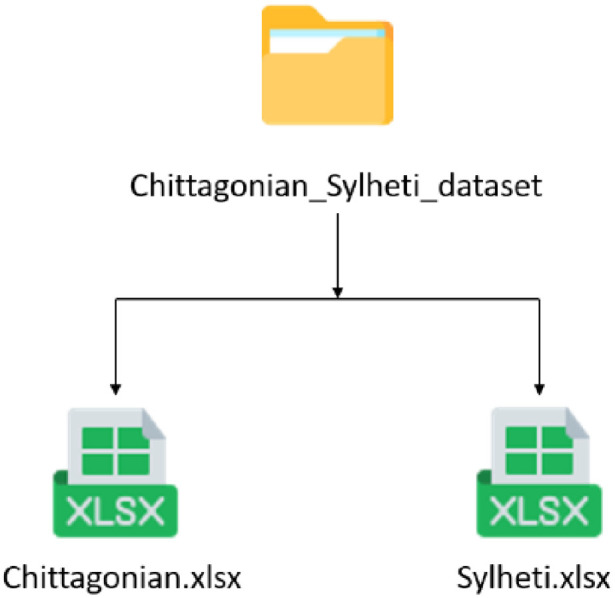
File structure of dataset.

The parallel corpora for Chittagonian-Bangla and Sylheti-Bangla translation are kept in two separate XLSX files (Fig. 1). Each file includes three columns: Standard Bangla, Translated English and the corresponding dialect. The English translations were produced using Google Cloud Translation API [9], MyMemory Translation API [10], and the deep-translator Python library [11] from the Standard Bangla column to support wider understanding of the dataset. All translations were manually reviewed for errors and corrected where necessary. English translations are included to ensure wider accessibility and understanding. Since most NLP models are primarily trained on English, translating the dialect into English makes it easier to apply and evaluate a variety of NLP tasks.

The Chittagonian-Bangla corpus consists of 8,000 unique sentences, each aligned with its Standard Bangla equivalent, which contains around 13,200 distinct words (Table 1). Similarly, the Sylheti-Bangla corpus includes 9,300 unique sentences in the Sylheti dialect matched with their Standard Bangla counterparts (Table 2), featuring approximately 14,500 different words.

**Table 1:** A sample of the Chittagonian-Bangla diale*ct from* Chittagonian.xlsx *file.*

Standard Bangla	Translated English	Chittagonian Dialect
চাটগাঁইয়া বৃহত্তর চট্টগ্রাম অঞ্চলের আঞ্চলিক ভাষা।	Chittagonian is the regional language of the greater Chittagong region.	চাটগাঁইয়া ডঅর চাটগাঁ অঞ্চলর আঞ্চলিক ভাষা।
চাটগাঁইয়া মূলত মৌখিক ভাষা।	Chittagonian is primarily an oral language.	চাটগাঁইয়া আদতে মুখর ভাষা।
এর নিজস্ব লিপি পদ্ধতি নেই।	It does not have its own script.	ইবা নিজর মতো লেকনর কন কুদরত নাই।
তাঁকে ধন্যবাদ ও শুভেচ্ছা জানাই।	Thank you and congratulations to him.	তাঁরারে ধইন্যবাদ আর শুবেচ্ছা জানাইর।
আপনি কী ধরনের ব্যবসা করেন?	What kind of business do you do?	অনে কন ডইল্যা বেয়ারি?

**Table 2:** A sample of the Sylheti-Bangla dialect from Sylheti.xlsx file.

Standard Bangla	Translated English	Sylheti Dialect
এখানে কাছাকাছি হাসপাতাল কোথায় আছে?	Where is the nearest hospital?	ইখানো ধারো হসপিটাল কোয়াই আছে?
স্ত্রী কন্যাদের নিয়ে শ্বশুরবাড়ি যাচ্ছি।	I am going to my in-laws' house with my wife and daughters.	বউ ফুরিরারে লইয়া হওরবাড়িত যাইরাম।
কাছ থেকে দেখেছি।	I saw it up close.	ধার থাকি দেখছি।
রূপকথা শুনবেন?	Would you like to listen to a fairy tale?	কিচ্চা ফুনতায় নি?
তবে সে মোটেই ঘাবড়ায় নি।	But he wasn't nervous at all.	তবে হে এখটু ও ডরাইছে না।

**Fig. 2:**
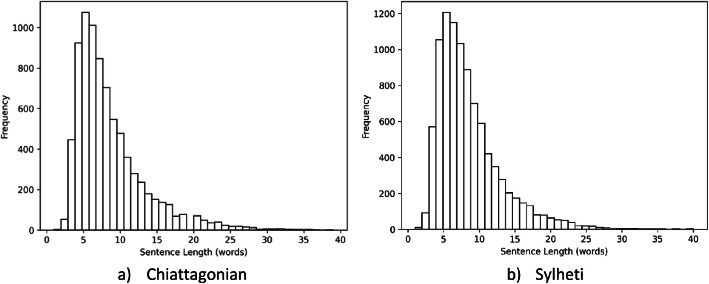
Sentence length distribution of dialect datasets.

**Fig. 3:**
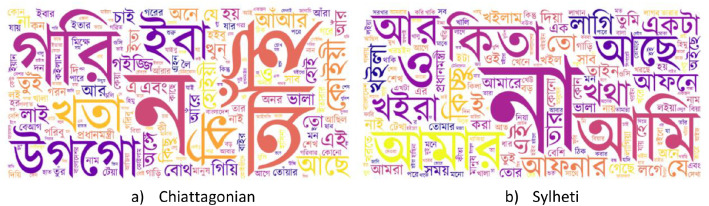
Snapshot of common words in dialect datasets*.*

The sentence distributions of Chittagonian and Sylheti dialects are shown in the Fig. 2. The dataset contains both long sentences of approximately 35–40 words and very short sentences consisting of only 1–3 words. However, the majority of sentences fall within the range of 5–10 words. Most common words of both dialects are also shown in Fig. 3.

**Fig. 4:**
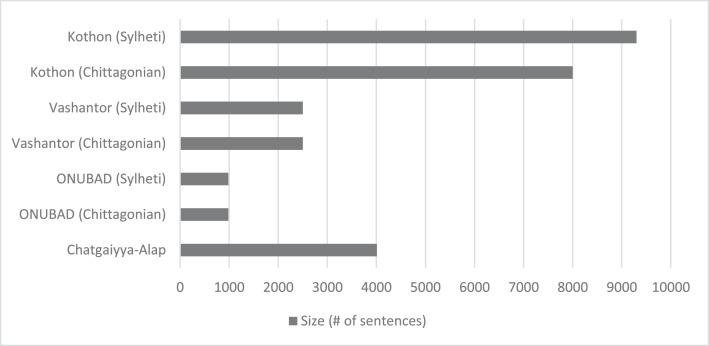
Comparison between datasets in terms of sentence length.

**Fig. 5:**
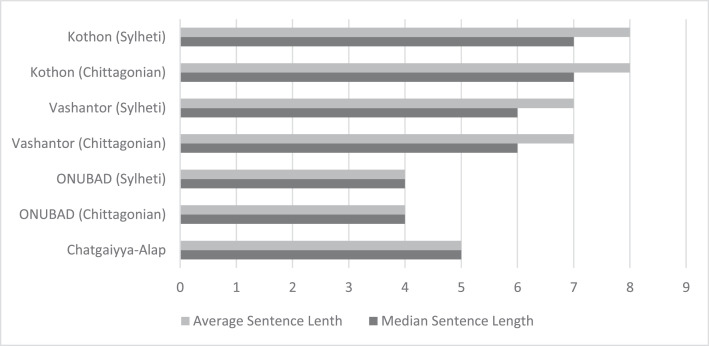
Comparison between datasets in terms of median and average sentence length.

## 4 Experimental Design, Materials and Methods

### 4.1 Dataset preparation

**Fig. 6:**
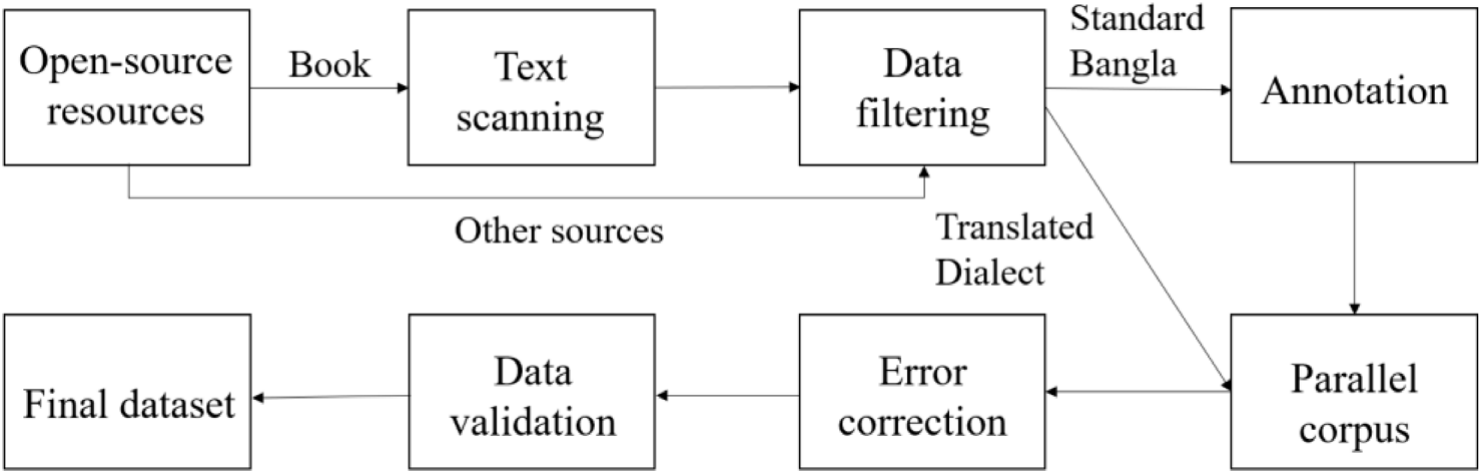
Dataset preparation process.

We collaborated with 5 annotatorswho are native Chittagonian and Sylheti speakers (Tables 3 and 4). The dataset preparation process is shown in Fig. 6.

1. **Open-source resources:** We used different open-source resources to build the dataset. Most Standard Bangla sentences were collected from novel books such as “হিমুর রূপালী রাত্রি” [12], “আজ হিমুর বিয়ে” [13] written by Humayun Ahmed, to capture natural conversations from daily life. In addition, some standard Bangla sentences were taken from the RisingNews dataset [14]. Around 500 pairs of Chittagonian and Standard Bangla sentences were collected from the book ”চাটগাঁইয়া সঅজ পন্না” written by Dr. Mahbubul Haque [15]. Several Chittagonian and Sylheti sentences were also gathered from open online platforms [16,17].
2. **Text scanning:** Since part of the data was available only in printed form, Google Lens [18] was used to scan text from books. The scanned content was saved as text files for later processing.
3. **Data filtering:** Most of the raw data was in paragraph format, so regular expressions were applied to split it into individual sentences. The sentence-splitting process was performed using the pattern r'[^।!?]*[।!?]'. Then we manually reviewed each sentence and irrelevant or duplicate sentences were then removed, and the filtered data was stored in XLXS files.
4. **Annotation interface:** For annotation, a web-based interface was developed using the Gradio library [19] and deployed on Hugging Face [20] (Fig. 7). Annotators used this tool to input their translation of Standard Bangla sentences into their respective dialects. We also added a comment field in the interface to collect additional information about translation.

**Fig. 7:**
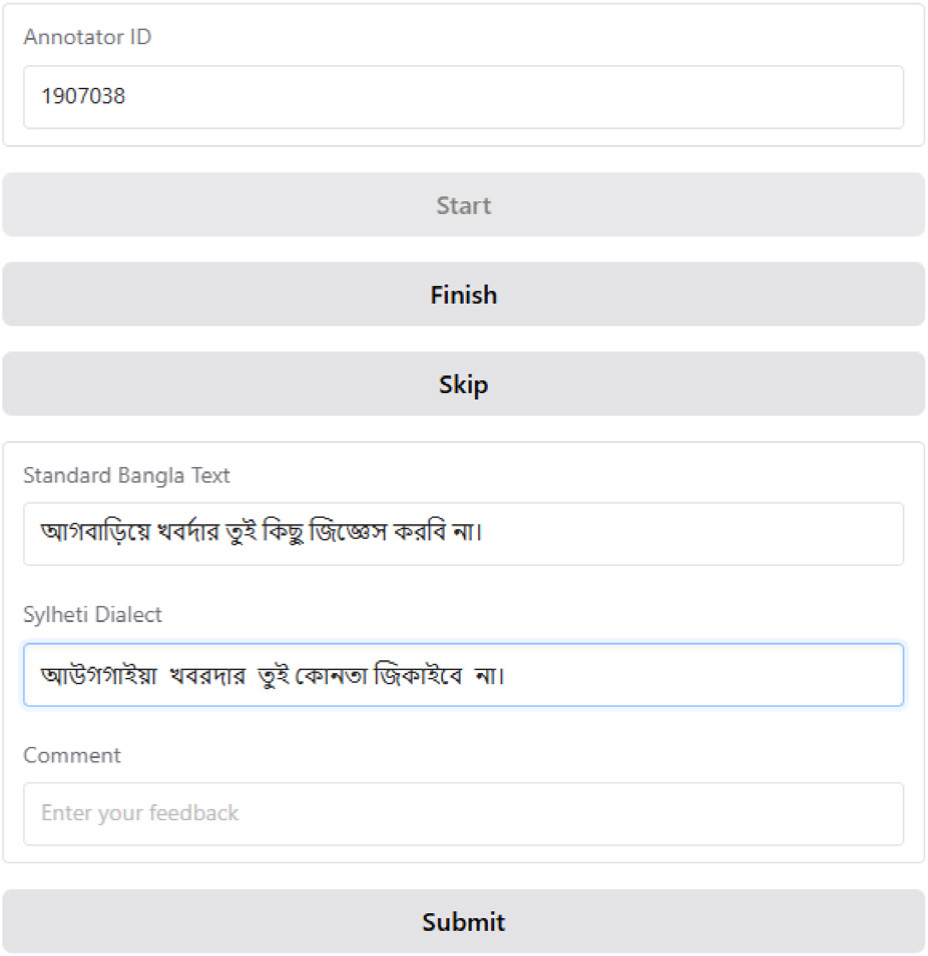
Data collection web app interface.
5. **Error correction:** To improve data quality, fuzzy clustering (Algorithm 1) was applied for each dialect. We used the RapidFuzz (version 3.13.0) library in Python 3.10 to compute similarity scores between words. For each word pair, the Levenshtein similarity was calculated using rapidfuzz.fuzz.ratio(w1, w2). This technique helped identify closely related words and highlight irrelevant or misspelled terms. Based on this clustering, incorrect entries were removed manually.

**Algorithm 1:** Group Similar Words.

**Require:** A list of *words*, similarity *threshold* (default = 80)	
**Ensure:** Groups of similar words	
1:	Initialize an empty list *groups*
2:	Initialize an empty set *visited*
3:	**for** each *word* in *words* do
4:	**if***word* ∈ *visited***then**
5:	continue to next word
6:	**end if**
7:	Initialize *group* ← [*word*]
8:	Add *word* to *visited*
9:	**for** each *other_word* in *words***do**
10:	**if***other_word* ∈*/ visited***and***fuzzy_ratio* (*word, other_word*) ≥ *threshold***then**
11:	Append *other_word* to *group*
12:	Add *other_word* to *visited*
13:	**end if**
14:	**end for**
15:	Append group to groups
16:	**end for**
17:	**return***groups*
6. **Final dataset:** After completing validation, the final dataset was stored in a separate XLSX file.

**Table 3:** Comparison between the Kothon, ChatgaiyyaAlap, ONUBAD, and Vashantor datasets.

Dataset	Dialect Coverage	Size (# of sentences)	Data Collection, Processing & Validation	Intended Applications
Chatgaiyya-Alap [6]	Chittagonian	4012	**Source:** Social media platforms (e.g., YouTube, Facebook) **Preprocessing:** Removal of numbers, emojis, and punctuation. **Translation:** Produced using a custom dictionary developed with professional translators. **Validation:** Evaluated by five professional native Chittagonian speakers, with additional individual reviews by multiple native speakers.	Machine translation, intelligent language systems, text-to-speech dialect conversion.
ONUBAD [8]	Chittagonian, Sylheti, Barishal, English-translated Standard Bangla	980 for each dialect	**Source:** Websites, books, and regional volunteers. **Translation:** Performed by skilled specialist translators with regional knowledge. **Validation:** Conducted by regional specialists and reviewed by Bangla lecturers and university professors.	Machine translation, linguistic analysis tool development, communication support for farmers, inclusive educational tool creation.
Vashantor [7]	Chittagonian, Sylheti, Barishal, Noakhali, Mymensingh	2500 for each dialect	**Source:** Social media, websites, and newspapers. **Translation:** Conducted by specialists with regional expertise. **Validation:** Inter-Annotator Agreement (IAA) applied using Cohen’s Kappa and Fleiss’s.	Machine translation, regional dialect detection model development.
**Kothon**	Chittagonian, Sylheti, English-translated Standard Bangla	Chittagonian: 8000; Sylheti: 9300	**Source:** Novels, scanned texts from books, and websites. **Preprocessing:** Regex-based filtering, fuzzy clustering for spelling normalization. **Translation:** Conducted by native speakers using a custom annotation UI. **Validation:** Multi-stage validation by native speakers; random sample reviews by independent speakers per dialect; manual verification of translation accuracy.	Machine translation, text-to-speech and speech-to-text systems, dialect identification, automatic speech recognition, communication support for tourists.

**Table 4:** Annotators’ background.

Age	Annotated Dialect	Location	Level of Education
24	Chittagonian	Chattogram	Undergraduate Student
24	Chittagonian	Sitakund	Undergraduate Student
24	Sylheti	Sunamganj	Undergraduate Student
22	Sylheti	Moulvibazar	Undergraduate Student
23	Sylheti	Sylhet	Undergraduate Student

### 4.2 Dataset validation

**Fig. 8:**
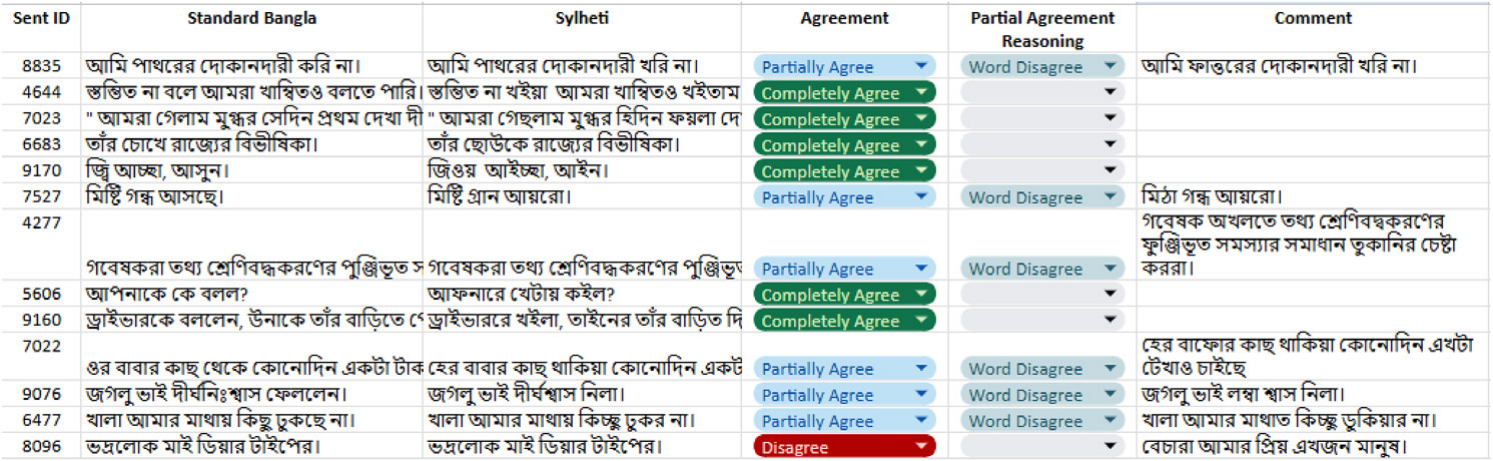
A snapshot of the validation configuration*.*

To ensure the quality and reliability of the collected dataset, we conducted a small validation experiment with the assistance of native dialect speakers who didn't partake in dataset annotation. As the corpus is quite large and we had a limited number of validators who were not involved in the translation process, we were only able to validate a small sample of the prepared dataset. This approach, however, ensured that the validation remained reliable and unbiased. Three random samples of 100 unique sentences were prepared from each dialect dataset. Each sample contained Standard Bangla and the corresponding dialect translation. The validators reviewed the translations thoroughly and scored them in three predetermined categories: *Completely Agree, Partially Agree*, and Completely *Disagree* (Table 5). Completely Agree indicated that the dialect translation was absolutely correct. A *Partially Agree* rating meant that the translation was mostly accurate but had minor issues including *Spelling Error*, and *Word Disagree*. Validators were instructed to mention the *Spelling Error*, or *Word Disagree* as the cause of the partially disagree case. Validators were also asked to note why they chose partially agree or disagree. A snapshot of the validation process is shown in Fig. 8.

**Table 5:** Validation category description.

Review Category		Description
*Completely Agree*		Translation is accurate.
*Partially Agree*	*Spelling Error*	Minor spelling error.
	*Word Disagree*	The validators did not fully disagree with the translation, but they prefer alternative wording for one or two specific terms.
*Completely Disagree*		Validators completely disagree with the translation.

In total, 300 samples are evaluated for each dialect. There is a significant amount of word disagreement (Table 6) in our dataset means that validators prefer different words. There is very little disagreement among the validators for our prepared dataset. Based on the comments of validators, we again reviewed our dataset and made corrections where required. After this rigorous validation process final dataset is prepared.

**Table 6:** Validation agreement result.

Dialect	Completely Agree	Partially Agree		Completely Disagree
		Spelling Error	Word Disagree	
**Chittagonian**	66.66%	2.67%	30%	0.67%
**Sylheti**	72%	3.67%	22.67%	1.66%

## Limitations

During validation, we observed a substantial number of word translations where validators expressed disagreement, not because the dataset was incorrect, but they preferred alternative word choices (Table 7, Table 8). This indicates that dialectal variations often differ across regions and even among individuals, particularly in the absence of standardized written forms for these dialects. Though our dataset represents the largest collection of Chittagonian and Sylheti dialects to this date (Figs. 4 and 5), it remains insufficient to cover the full variation of sentences used by native dialect speakers. Future work should focus on expanding these datasets, incorporating our rigorous validation standard and expanding to more diverse linguistic expressions and contexts.

**Table 7:** Some examples of Chittagonian word variations.

English	Standard Bangla	Chittagonian Variations
I did	করেছিলাম	কইরগিলাম, গইজ্জিলাম, গইরগিলাম
With	সাথে	অঙ্গে, লগে, ওয়াঁরে
Girl	মেয়ে	মাইয়্যাফুয়া, মাইয়্যা
Said	বললেন	কোইয়ী, হোইয়ী, কইলেন, হোইলেন
Pond	পুকুর	পইর, দইজ্জা

**Table 8:** Some examples of Sylheti word variations.

English	Standard Bangla	Sylheti Variations
Travel	ভ্রমণ করা	ফাকানিত যাওয়া, গুরানিত যাওয়া
Boy	ছেলে	ফোয়া, ফুত
Take a bath	গোসল করা	বোর ফারা, গতর ধোয়া
Not entering	ঢুকছে না	ঢুকর না,ডুকিয়ার না
Have to do	করতে হয়	খরতে ওয়, খরা লাগে

## Ethics Statement

This study does not include any human or animal experiments. All the data used in this work were gathered from open-source books and publicly available websites. These sources are freely accessible and were used according to their open licenses and terms of use. Proper references and acknowledgments have been provided where necessary. The authors confirm that the collection and use of these open-source materials comply with all ethical standards for research and publication.

## Credit Author Statement

**Md. Atique Faisal:** Conceptualization, Writing - Original Draft, Methodology, Data curation, Investigation, Validation; **Farhan Sadaf:** Conceptualization, Writing - Review & Editing, Formal analysis, Project administration, Supervision; **Dipta Chowdhury:** Data Curation, Validation, Investigation. **H.M. Azrof:** Data Curation, Validation, Investigation; **Monojit Paul Tanmay:** Data Curation, Validation, Investigation.

## Data Availability

Mendeley DataKothon: A Large-Scale Dataset for Machine Translation of the Chittagonian and Sylheti Dialects into Standard Bangla (Original data).
